# Moving From Organ Donation to Knowledge Donation: A Novel Opportunity for Surgical Education Following Organ Donation

**DOI:** 10.7759/cureus.10473

**Published:** 2020-09-15

**Authors:** Peter Wu, Rebecca Rieger, Margaret M McBride, Kayla Gray, James Mankin

**Affiliations:** 1 Surgery, St. Joseph's Hospital and Medical Center, Phoenix, USA; 2 Mission Integration, St. Joseph's Hospital and Medical Center, Phoenix, USA; 3 Research Projects Coordinator, Donor Network of Arizona, Phoenix, USA

**Keywords:** organ donor, organ procurement, organ harvesting, organ recovery, knowledge donation, surgical education, organ retrieval, organ donation

## Abstract

The objective of this article is to share how our institution implemented the use of organ donors for surgical education following organ recovery. Despite technological advances, realistic surgical simulation models are lacking, leaving little opportunity to practice a procedure prior to performance on a living patient. Utilization of organ donors following organ donation offers an opportunity for life-like surgical simulation. We developed a pathway to use organ donor tissue in the post-recovery period for robotic simulation. We obtained support from our local Institutional Review Board, Ethics Committee, and organ procurement organization to create a “knowledge donor” program. Our knowledge donation program provided learners hands-on experience with a novel procedure and also provided organ donors another opportunity to express their altruism. We found that the process was well accepted by donor families and learners. We implemented a knowledge donation program at our hospital that provides valuable surgical experience. We discuss future directions for knowledge donation at our institution.

## Introduction

Cadaver dissection for the purpose of education has a long and rich history in medicine [1]. For centuries, dissection of the human body was considered sacrilegious. Anatomical teachings were based on the dissection of animals and extrapolated to humans. Thus, many misconceptions about human anatomy and physiology were passed down through generations of physicians. Cadaver dissections were rare and typically performed on criminals who had been sentenced to death, contributing to public perception of dissection of the human corpse as a form of punishment [2]. Once the social and religious taboos surrounding cadaver dissection faded, exploration of the human body blossomed. During the Renaissance, artists, academics, and physicians alike began the detailed study of human anatomy, and cadaver dissection became an acceptable practice. Consequently, the demand for cadavers rose, leading to nefarious acts to procure cadavers such as grave robbing [3]. Cadaver dissection was ultimately incorporated into medical training for the purpose of teaching anatomy. Medical students at these pioneering medical schools were obligated to pay for and attend the funerals of their cadavers. Today, the tradition of cadaver dissection in the United States relies on the generosity of donors [4] and remains at the core of medical education. The study of human anatomy through cadaver dissection is often regarded as a rite of passage to the practice of medicine.

In modern times, the provision of practical, “hands-on” training to medical students, residents, and accomplished physicians alike faces shared challenges. Medical student education in the clinical years seems to be shifting away from opportunities for direct experience with procedures towards more of an observational experience. In residency training in the United States, direct patient care is limited by duty hour restrictions, increasingly burdensome clerical duties, and a litigious society in which patients may be hesitant to receive direct care from trainees. Thus, to maximize training, there is an increased reliance on simulation to provide deliberate practice opportunities in an environment that does not pose the potential for patient harm [5]. There are a multitude of simulators available for procedural tasks; however, the experience of a simulated procedure is only as good as the simulator itself [6]. After completing formal training, surgeons must keep pace with the rapid development of new procedures and technologies but have limited opportunity to do so in a hands-on fashion [7]. As novel techniques and devices are introduced, there is a growing need among practicing surgeons to refine existing skills or acquire new skills in a safe, realistic, affordable, and accessible fashion [8-10]. Many participate in cadaver labs to explore new surgical techniques and devices [11]. Cadavers, however, are expensive, and often physicians need to travel considerable distances and take time away from their practices to gain these types of experiences.

At our institution, we have begun to address these challenges through a novel use of organ donors following organ recovery. This developed as a byproduct of our exploration of performing robotic nipple-sparing mastectomy. Robotic mastectomy is gaining traction in Europe and Asia, but the surgical robot has yet to be approved for use in mastectomy in the United States. Despite careful study of the procedure, observerships overseas, and discussion with the world’s experts in robotic mastectomy, we were still left with no actual experience in performing the procedure ourselves. We felt that in order to offer future patients a safe and efficient robotic mastectomy with the best possible outcomes, we were obligated to seek direct, hands-on experience. There is currently no simulator or course available to train surgeons to perform robotic mastectomy. We sought the opportunity to perform robotic mastectomy on organ donors in the immediate post-recovery period in the operating room (OR).

## Technical report

As we were proposing to use non-living subjects, we first confirmed with our Institutional Review Board (IRB) that we were exempt from IRB oversight. Next, we engaged our Ethics Committee, which included our institution’s religious leadership. From an ethics standpoint, there were no objections to our proposal so long as the process involved obtaining formal consent from the patient’s family, full disclosure and complete transparency regarding the intended procedure, and lack of conflicts of interest. We then proposed the process to the local organ procurement organization (OPO) and hospital administration, which included executive, OR, critical care, and nursing leadership.

From a regulatory standpoint, the procedure took place under the purview of the OPO’s research and training protocols. We formalized a consent form for the procedure that was included as an addendum to the OPO’s research and training consent forms. There was a unanimous agreement that the normal processes of organ donation would take priority over any proposed post-recovery intervention. During the planning phase, our goal was to minimize disruption of the established organ donation process as well as the routine functioning of the OR while providing the necessary learning opportunity for the robotic mastectomy team. Thus, we agreed to a 90-minute window immediately following organ recovery during which our surgical team would have the opportunity to perform robotic mastectomy and reconstruction. This time limit would also serve to limit resource utilization and contain cost. All costs were to be borne by the hospital. The robotic mastectomy and reconstruction would be performed with standard sterile technique so that the donor may proceed to additional tissue donation and research.

From a donor and donor family’s perspective, we provided inclusion and exclusion criteria for suitable candidates to the OPO. When a potential candidate was identified, robotic team personnel and OR availability would be confirmed. The OPO’s donor family advocate would counsel the family regarding the educational intent of post-recovery robotic mastectomy with reconstruction. The family was also offered the option to discuss the procedure further with our surgical team. Any specific accommodations or limitations to our intervention requested by the family would be honored. Since donation was considered an anatomical gift, participation was completely voluntary and could be withdrawn at any time. There were no financial incentives offered to the patients or their families to participate.

Organ donors were considered for robotic mastectomy starting in March 2019. From March to September 2019, there were 361 in-hospital deaths, of which 20 (5.5%) were of organ donors. Five (25%) of these organ donors met our criteria, and their families were approached for robotic mastectomy. All five donors consented to participation. Among those consented, two proceeded with robotic mastectomy immediately following organ recovery. Concerning the three candidates who did not undergo robotic mastectomy, one was excluded from participation due to OR time constraints and the remaining two were to undergo organ donation following cardiac death, but neither expired within the allotted period after terminal extubation to allow for procession to organ donation.

The first donor who underwent robotic mastectomy was a 23-year-old registered organ donor with a family history of breast cancer who died of drug overdose. After cardiac death, kidneys were retrieved for organ transplantation, and liver and ovaries were retrieved for research. Immediately following organ donation, our team proceeded with a unilateral robotic mastectomy in 90 minutes without reconstruction. Following robotic mastectomy, skin, cartilage, tendon, and bone for allograft tissue transplantation were retrieved in a customary fashion under the purview of the OPO.

The second donor was a 66-year-old non-registered organ donor who worked as a medical provider and sustained an irrecoverable head injury. Kidneys were retrieved for organ transplantation and liver for research. Immediately following donation, our team proceeded with bilateral robotic mastectomy and unilateral implant reconstruction in 90 minutes. Additional tissues were not recovered.

## Discussion

The benefits of practicing robotic mastectomy on a fresh cadaver in the post-recovery phase of organ donation were immediately evident. First and foremost, organ donor tissue is the highest fidelity “simulator” available, offering an experience secondary to that of operating on a living patient. The tissue handling characteristics are indistinguishable from a living patient with the exception of lack of normal bleeding and lack of chest wall respiratory excursion. In contrast, the tissue quality of traditional cadavers leaves much to be desired in terms of how closely it resembles living tissue and would not have provided a realistic operative experience.

Secondly, the vast majority of breast surgeons have little to no robotic experience, and simulated robotic mastectomy is not available. Even for recent graduates who receive formal robotic training during surgical residency, there is concern that for many this experience is insufficient [12]. Thus, hands-on training experiences for those interested in learning robotic mastectomy are non-existent. Our team felt ethically and morally compelled to become thoroughly familiar with the procedure before embarking on a robotic mastectomy clinical trial.

Logistically, performing robotic mastectomy immediately following organ recovery in the OR was advantageous as trained OR staff and all of the necessary equipment were immediately available. This degree of robotic expertise and support is unavailable in most cadaver labs, including our own. At our institution, the surgical robot is not available in the cadaver lab, and acquiring a new robot or temporarily relocating an existing robot would take tremendous resources.

From a cost perspective, the extra 90 minutes of OR time represents a cost that, in our hospital, has been calculated to be approximately $4,500; however, this is largely an indirect cost. This is similar to the cost of a fresh frozen cadaver torso. Comparing cost in this manner does not reflect the true value of performing robotic mastectomy in the OR. As previously mentioned, having the most realistic training experience is invaluable. The OR also offers a higher fidelity experience to the actual robotic mastectomy with respect to room layout, patient placement, robot placement, and docking, as well as the availability of familiar surgical instruments and supplies, rather than relying on whatever is available in the cadaver lab. Furthermore, time in the OR with trained robotic personnel is more effective than the same amount of time spent in our cadaver lab where the staff are not familiar with the surgical robot.

Our first robotic mastectomy experience led to significant changes in our technique compared to what we had observed overseas. We made adjustments related to patient positioning, robot docking, and instrumentation. We were able to assess the portions of the procedure that were likely going to be more difficult and where we might encounter complications. We were able to anticipate potential intraoperative complications and their management in real time. Furthermore, the experience allowed us to introduce the procedure to OR staff so that they may gain some familiarity prior to performing the procedure on an actual breast cancer patient.

After applying what was learned from the first experience and implementing changes, our second donor experienced encompassed bilateral robotic mastectomy and a unilateral implant reconstruction in the same amount of time that we took to perform our first unilateral robotic mastectomy. Our experience with the second donor solidified the familiarity of the procedure among the OR staff and prompted significant feedback in real time that allowed us to further improve our technique. Additionally, the robotic team became more invested in the procedure, and this improved team dynamics and communication.

There are some limitations to the use of organ donor tissue. The anatomical area of interest may be altered by disease, injury, or the recovery process itself. Another disadvantage to using organ donors is that the timing of the learning activity is not scheduled in advance and commonly occurs at inopportune times. The organ donation process requires a tremendous degree of coordination among several facilities, potential organ recipients, and surgical teams. Every effort was made to avoid any negative impact to this process by our learning activity, and thus we accepted our opportunities whenever they became available.

We believe that despite these limitations, the use of organ donor tissue in this manner has the potential to impact medical training and surgical education in a significant manner. To our knowledge, the immediate use of organ donor tissue for medical education purposes is novel and not previously described. The proposal to perform post-donation interventions for the purposes of education and/or research, however, is a natural and logical extension of the patient’s and family’s desire to help others. A single donated organ provides direct benefit to a single recipient, but when knowledge is donated, innumerable future patients benefit. When framed in this way, it was easy to understand why the families of the two organ donors were not only agreeable to robotic mastectomy, and they expressed that they felt that the gift of knowledge was more important than the organ donation itself.

Once agreeable to organ donation, we suspected that most would also consent to post-donation learning activities for innovation or research. Indeed, none of the donors approached for robotic mastectomy declined the opportunity. This is consistent with studies suggesting that most patients and families would agree to post-mortem training procedures for themselves or a family member [13-15].

We are currently developing a program to further expand the pool of knowledge donors. Half of all Arizona residents are registered organ donors; however, of the nearly 700 deaths at our hospital each year, fewer than 10% proceed to organ donation. We propose to offer families of dying or recently deceased hospitalized patients (with some medicolegal and infectious disease exceptions) the opportunity to make an anatomical gift to medical science even if they are ineligible for organ donation. Although there is some similarity to other established programs, this concept would be the first of its kind. First, we propose to recruit through voluntary participation and informed consent. Despite most authors agreeing that consent is necessary [16-18], there are other programs in existence that use the bodies of unclaimed deceased for educational purposes or who conduct informal, impromptu learning activities on newly deceased patients without consent [19,20]. Another distinguishing characteristic of our program is the provision of fresh, non-frozen, non-preserved tissue to simulate the most realistic learning experience possible.

## Conclusions

Based on our experience, we believe that knowledge donation immediately following organ donation is both feasible and useful, offering a learning platform that nearly replicates an operation on a living patient. Additionally, we speculate that families of recently deceased patients who die in the hospital would support a knowledge donor program to educate medical learners and help physicians improve patient care even if they are not proceeding with organ donation. We acknowledge the ethical concerns regarding the use of recently deceased individuals for scientific and educational purposes, but this concept of anatomical donation to science is not novel. National programs such as the United Tissue Network and Science Care facilitate the donation of tissue to research and educational endeavors. Our proposal for a knowledge donor program at our institution would provide this same opportunity on a local scale with the intent of improving education and patient safety but with the advantage of yielding life-like tissue. Furthermore, knowledge donation and whole-body donation are not mutually exclusive. As we demonstrated with our organ donor experience through thoughtful preparation and careful communication with the OPO, patients can be organ donors, participate in learning activities as knowledge donors, and also proceed with tissue donation for clinical and research use. Clear communication with the family, careful planning of learning activities, and coordination with OPO’s and whole-body donation programs would maximize the yield of a generous anatomical gift while adhering to the principle of patient autonomy.

Nationwide, there are roughly 2.5 million hospital deaths per year, of which less than 1% proceed to organ donation. Knowledge donation is a voluntary process that provides a legal, ethical, and respectful means to address some of the challenges of medical education and surgical training. Knowledge donation provides another opportunity to honor the wishes of patients and can be performed alongside organ donation, tissue donation, and/or whole-body donation. If knowledge donor programs became more common, we believe that patient safety would improve, medical education would flourish, and surgical innovation would blossom.
